# Ecological release from interspecific competition leads to decoupled changes in population and individual niche width

**DOI:** 10.1098/rspb.2010.0018

**Published:** 2010-02-17

**Authors:** Daniel I. Bolnick, Travis Ingram, William E. Stutz, Lisa K. Snowberg, On Lee Lau, Jeff S. Paull

**Affiliations:** 1 Section of Integrative Biology, University of Texas at Austin, Austin, TX 78712, USA; 2 Department of Zoology and Biodiversity Research Center, University of British Columbia, 6270 University Boulevard, Vancouver, British Columbia, CanadaV6T 1Z4

**Keywords:** ecological release, *Gasterosteus aculeatus*, individual specialization, interspecific competition, niche variation hypothesis, three-spine stickleback

## Abstract

A species's niche width reflects a balance between the diversifying effects of intraspecific competition and the constraining effects of interspecific competition. This balance shifts when a species from a competitive environment invades a depauperate habitat where interspecific competition is reduced. The resulting ecological release permits population niche expansion, via increased individual niche widths and/or increased among-individual variation. We report an experimental test of the theory of ecological release in three-spine stickleback (*Gasterosteus aculeatus*). We factorially manipulated the presence or absence of two interspecific competitors: juvenile cut-throat trout (*Oncorhynchus clarki*) and prickly sculpin (*Cottus asper*). Consistent with the classic niche variation hypothesis, release from trout competition increased stickleback population niche width via increased among-individual variation, while individual niche widths remained unchanged. In contrast, release from sculpin competition had no effect on population niche width, because increased individual niche widths were offset by decreased between-individual variation. Our results confirm that ecological release from interspecific competition can lead to increases in niche width, and that these changes can occur on behavioural time scales. Importantly, we find that changes in population niche width are decoupled from changes in the niche widths of individuals within the population.

## 1. Introduction

The niche width of a species is generally thought to reflect a balance between the diversifying force of intraspecific competition and the constraining effect of interspecific competitors (Van Valen 1965; Roughgarden 1972; Grant & Price 1981; Taper & Case 1985). When the relative strengths of these forces change, niche width should change accordingly. For example, niche expansion is commonly observed when species from highly competitive environments invade species-poor habitats with fewer interspecific competitors, such as oceanic islands or post-glacial lakes (Van Valen 1965; Diamond 1971; Kohn 1978; Feinsinger & Swarm 1982; Dayan & Simberloff 1994; Robinson & Wilson 1994; Losos & de Queiroz 1997; Persson & Hansson 1999; Robinson *et al*. 2000). This niche expansion, known as ‘ecological release’, occurs because the invading species can access resources that may have otherwise been depleted or monopolized by competitors.

During ecological release, newly available resources may be added to the population's niche for any of several reasons. First, niche expansion may be non-adaptive: in the absence of competitors, stabilizing selection on resource use may be relaxed, thereby allowing increased frequency of neutral or slightly deleterious resource-use phenotypes. Second, niche expansion may be adaptive if the newly available resources are inherently more valuable than previously used resources. Third, the new resources may be inherently less profitable than previous resources, but niche expansion may still be favoured by density- or frequency-dependent selection (Wilson & Turelli 1986; Svanbäck & Bolnick 2005, 2007). Increased intraspecific competition can deplete the population's usual resources to the point where normally low-value novel resources are relatively profitable (Bolnick 2001, 2004; Martin & Pfennig 2009; Svanback & Persson 2009).

### (a) Individual versus population release

Most definitions of the niche focus on the ecological interactions of a population or species as a whole (Hutchinson 1957; Schoener 1989; Chase & Leibold 2003). Instead of this typological view of the niche, we argue that the niche is an emergent property of individuals’ phenotypes and hence should be defined at the level of the individual. The population's niche is thus an aggregate of the biotic or abiotic interactions experienced by potentially heterogeneous individuals. For instance, the hunting wasp *Trypoxylon albonigrum* consumes at least six genera of spiders, but any individual specializes on only one or two spider genera (Araújo & Gonzaga 2007). Such ‘individual specialization’ has been documented in well over 100 species (Bolnick *et al*. 2003).

**Figure 1.**
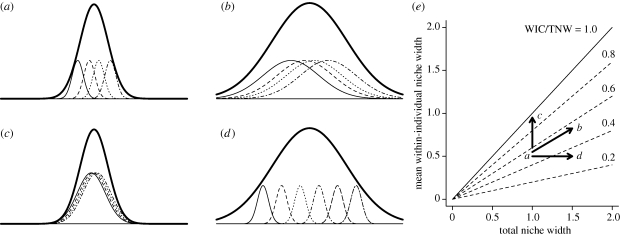
Illustration of three potential forms of ecological release. Consider a population that (*a*) initially coexists with an interspecific competitor; the population niche width is indicated by a thick curve, and niche widths of four individuals are indicated by shorter, thin lines. Release from competition can lead to (*b*) increased individual and population niche widths (parallel release); (*c*) increased individual but not population niche widths, because expansion is offset by decreased among-individual variation (individual release); or (*d*) increased population but not individual niche widths, via increased among-individual variation (niche variation hypothesis). Each of these scenarios (*b*–*d*) is plotted as (*e*) a vector in a niche space graph. In this type of graph, we plot individual versus population niche width (WIC versus TNW). Because TNW = WIC + BIC, any population must fall on or below the solid line where WIC/TNW = 1. Thin dashed lines below this represent increasing isoclines of individual specialization (smaller WIC/TNW). Ecological release can be plotted as a vector in this space, from the high to low competition niche widths.

In the following paragraphs, we outline three distinct patterns of ecological release that are made possible by this decoupling of individual and population niche widths. Before we outline these alternatives, we need to define a few terms. Consider a population that uses a set of resources that vary in some quantitative trait (e.g. size). The population's total niche width (TNW) is simply the variance of the size of all prey used by all members of the population. This TNW has a within-individual component (WIC) and a between-individual component (BIC) such that TNW = WIC + BIC (Roughgarden 1972). The WIC is the average variance in prey sizes used by a typical individual, while the BIC is the variation among individuals’ mean prey sizes (figure 1). Equivalent measures of TNW, WIC and BIC can be obtained for categorical prey data using the Shannon diversity index as a substitute for variance (Bolnick *et al*. 2002).

The partitioning of TNW into within- and between-individual variation highlights two different paths by which population TNW can increase during ecological release. First, all individuals can increase their own niche width (WIC) by shifting to use a newly abundant high-value prey type. We refer to this as ‘parallel release’ (figure 1*b*) because individual and population niche widths change in similar ways. Parallel release is predicted by a number of adaptive dynamics and quantitative genetic models, which indicate that individuals should evolve to use the full range of the population's resources (WIC = TNW; Roughgarden 1972; Taper & Case 1985; Ackermann & Doebeli 2004).

The second pattern of ecological release is the ‘niche variation hypothesis’ (NVH; Van Valen 1965), in which TNW increases via greater between-individual variation (BIC) while individual niche widths remain constant (figure 1*d*). This may occur when functional trade-offs limit individuals’ ability to efficiently use multiple types of prey. For example, simple biomechanical rules for lever systems make it difficult to generate both large forces and high speeds with a given morphological structure, limiting the diversity of prey an individual forager can capture effectively (Wainwright 1996). Individuals also experience cognitive limits to the number of search images that can be maintained simultaneously (Persson 1985; Lewis 1986). Such trade-offs may place upper bounds on individual niche widths, such that population niche expansion can only occur if individuals diverge in resource use. Between-individual variation can increase if only some individuals shift to novel resources, or if different individuals adopt different novel resources (figure 1*d*). The NVH has received very mixed empirical support in the 45 years since it was first proposed (Soule & Stewart 1970; Rothstein 1973; Bernstein 1979; Meiri *et al*. 2005; Bolnick *et al*. 2007; Costa *et al*. 2008). In particular, studies focusing on morphology or size have often failed to find the predicted positive correlation between intraspecific trait variation and population niche width. Recent studies suggest that this tepid support is because morphological variance is a poor proxy for diet variation, for which data are more supportive of the NVH (Bolnick *et al*. 2007). This is because when two variables are moderately to weakly correlated (e.g. stickleback morphology and diet; Bolnick & Paull 2009), variance in one trait has little effect on variance in the second trait.

The two scenarios described so far correspond to the classical view of ecological release as increased population niche width. We can also envision a third form of ecological release (‘individual release’) in which TNW does not change. Consider a population in which some individuals specialize on prey A and ignore B, whereas others specialize on B and ignore A. During ecological release, individual niche widths may expand such that all individuals use both A and B. This individual niche expansion is offset by decreased variation among individuals, so the population niche remains unchanged (figure 1*c*). Individual release can occur if an initially heterogeneous population experiences reduced prey availability, inducing all individuals to begin using prey they previously ignored (in line with optimal foraging theory; Schoener 1971; Stephens & Krebs 1986). It is, of course, counterintuitive to think that ecological release could reduce prey availability, but this can arise if intraspecific competition increases disproportionately following release (Trewby *et al*. 2007), or if competitive release drives trophic cascades that indirectly reduce prey availability. Alternatively, if competitive release increases prey availability, individual niche width may expand for reasons not accounted for by optimal foraging theory (e.g. reduced interference competition, changes in predation risk, etc.).

To summarize, the parallel release, individual release and NVH make three contrasting predictions about responses to reduced interspecific competition (figure 1). Here, we report the results of a field experiment designed to (i) test whether removal of interspecific competitors leads to changes in population niche width and (ii) determine which form of ecological release takes place (parallel, individual or NVH). We tested for short-term changes in the trophic niche of the three-spine stickleback (*Gasterosteus aculeatus*) after experimentally manipulating the presence or absence of two interspecific competitors.

Stickleback exhibit substantial individual specialization within single populations in north temperate lakes (Svanbäck & Bolnick 2007; Araújo *et al*. 2008; Bolnick *et al*. 2008; Snowberg & Bolnick 2008; Matthews *et al*. in press). Even within morphologically unimodal and genetically panmictic populations, individuals tend to forage in different microhabitats (Bolnick *et al*. in review) and select different subsets of the available prey. Analyses of stomach contents indicate that, on average, two individuals picked at random share only approximately 30 per cent of their diet in common (Araújo *et al*. 2008; Bolnick & Paull 2009). Stable isotope analyses confirm that these diet differences between individuals are sustained over months (Bolnick *et al*. 2008). Diet differences are associated with variation in trophic morphology such as gill raker length or number (Bolnick *et al*. 2008; Bolnick & Paull 2009; Matthews *et al*. in press).

A recent experiment showed that stickleback are a promising system in which to study ecological release. Svanbäck & Bolnick (2007) manipulated stickleback density in 10 m^2^ enclosures in a natural lake population. The high-density treatment led to reduced zooplankton and benthic invertebrate availability, and corresponding reduction in stickleback stomach content mass and growth rates. Consistent with the NVH, strong intraspecific competition drove population niche expansion via greater between-individual variation while individual niche widths remained unchanged (figure 1*d*). Here, we expand on their previous study to explicitly test the effect of ecological release from interspecific competitors.

## 2. Material and methods

### (a) Experimental design

In late May 2007, we constructed 20 experimental enclosures in Blackwater Lake, on northern Vancouver Island, British Columbia (50.1993° N, 179.588° E). The approximately 10 m^2^ (3.3 × 3.3 m) enclosures were constructed of 1/16" seine netting arranged in five blocks of four enclosures distributed along the shoreline of the lake in water that ranged from 1 to 2 m deep. We used minnow traps and dipnets to remove any accidentally enclosed fish. Stickleback collected from similar habitat near the enclosures were mixed, then randomly divided among enclosures (*n* = 40 per cage). A prior experiment in the same lake found that a density of three fish per square metre led to growth rates and prey densities comparable with natural conditions (Svanbäck & Bolnick 2007). Thus, our treatment of four fish per square metre should impose moderately elevated intraspecific competition.

In each block of four enclosures, we factorially crossed the presence or absence of two potential competitor species, the prickly sculpin (*Cottus asper*) and the juvenile cut-throat trout (*Oncorhynchus clarki*). The four treatments were (i) competition (four sculpin, seven trout), (ii) release from sculpin (trout present), (iii) release from trout (sculpin present) and (iv) total release (no competitors). These competitor densities represent the upper end of natural levels, based on observations made by snorkel transects. Note that we interpret our experimental results in terms of the effect of ecological release from competition, but one could equally couch our discussion in terms of the effects of competitor addition (treating the no-competitor treatment as the base-line state). Trout and sculpin were caught locally using dipnets and minnowtraps. We used small sculpin or trout (<10 and <8 cm, respectively) to ensure that competition treatments were not confounded by intraguild predation on stickleback. Total biomass of sculpin and trout were approximately equal in the competition treatments, although total metabolic rates may be different owing to unequal body sizes.

Three lines of evidence suggest that these species compete with stickleback. First, observations by snorkelers confirm that both trout and sculpin regularly co-occur with stickleback in their shared feeding habitats, and stickleback direct attacks on sculpin, suggesting some degree of interference competition. Second, a comparative study of stickleback in lakes with versus without sculpin and/or trout found evidence of character displacement in trophic morphology, consistent with interspecific competition (R. Svanbäck & D. Schluter 2006, unpublished data). Finally, direct examination of stomach contents of sculpin and trout reveals overlap with stickleback diets (§3). Sculpin are exclusively benthic feeders, whereas juvenile trout feed at the surface and in the water column. In contrast, stickleback feed in both microhabitats, although any single individual tends to specialize on one or the other (Bolnick *et al*. in review). Consequently, release from sculpin and trout competition may have contrasting effects on stickleback feeding behaviour, both for individuals and for the population as a whole.

The experimental treatments were left undisturbed for 15 days, after which all stickleback were removed by dipnet or trapping within a 4 h period. Traps were checked every 2 h, to minimize effects on stomach contents (<6 h is sufficient to avoid bias; R. Svanbäck & D. I. Bolnick 2005, unpublished data). We also removed trout and sculpin but both species are evasive so recapture numbers were low. Specimens were immediately preserved in 10 per cent neutral buffered formalin. We weighed and sexed each fish, and measured standard length, gape width, gill raker length and gill raker number. We identified (to the lowest feasible taxonomic level) and counted prey in stomachs of each stickleback, trout and sculpin.

### (b) Quantifying niche breadth and diet variation

Within each enclosure, we used the frequencies of prey taxa in stickleback stomachs to calculate Shannon diversity measures of population TNW, and its WIC and BIC (see Bolnick *et al*. 2002 for equations). Shannon diversity reflects both the number of prey categories consumed and the evenness with which they are consumed. Hence, increased evenness can alter WIC and TNW even if no new prey types appear.

To measure individual specialization, we used the ratio of the average individual niche width to the population niche width (WIC/TNW). This ratio is a dimensionless index that ranges from one, when all individuals consume the same prey in the same proportions (no individual specialization), down to zero, when each individual uses a unique type of prey (maximal individual specialization). We used a Monte Carlo resampling routine to test whether the observed diet variation in each enclosure departed significantly from the null hypothesis that all individuals sample equally (but stochastically) from a single distribution of prey (for details, see Svanbäck & Bolnick 2007; Araújo *et al*. 2008). To confirm that our results are not sensitive to the particular measure of individual specialization, we calculated an alternative index, *E*, the mean pairwise diet dissimilarity between individuals (Araújo *et al*. 2008). We did not directly examine individual specialization within the trout or sculpin, owing to the small sample sizes initially placed in each enclosure, and the even lower recapture numbers. However, we did calculate the proportional similarity, PS (Schoener 1965), between stickleback and each competitor using the population diet composition of each species, aggregated across all enclosures.

### (c) Statistical analyses

#### (i) General effects of interspecific competition

To test whether release from interspecific competitors increased sticklebacks’ foraging success, we converted prey counts into an estimate of stomach content mass using published length–weight regressions. Similar results were obtained with total prey lengths and prey counts, but we report masses because they have more direct implications for energetic income. Gut fullness relative to fish size was measured as the residuals from a regression of total prey mass on body mass (both log-transformed, using all 825 stickleback guts). We then tested whether residual gut fullness differed consistently between competition treatments. As in all the following analyses, enclosures are the level of replication, so we averaged the residual gut fullness for all stickleback within an enclosure. We then used a mixed-model analysis of variance (ANOVA) to test for a random block effect, for effects of removal of sculpin or trout, and for an interaction between trout and sculpin removal, affecting residual gut fullness. We conducted mixed-model ANOVAs using the *lme4* package in R (R Development Core Team 2007). Statistical significance of fixed effects was determined by likelihood ratio test comparisons of successively simpler models, which agreed with Akaike Information Criterion (AIC) model selection methods. Normality of residuals was confirmed via quantile plots.

We tested whether changes in foraging success affected stickleback energy reserves. Successful foragers have a higher energy income, resulting in higher condition index, CI (the residuals of log-transformed body mass dependent on log standard length, sex and a length × sex interaction). A mixed-model ANOVA then tested whether CI depends on block and removal of sculpin, of trout, or their interaction.

#### (ii) Ecological release

To test for ecological release of stickleback TNW, we used a mixed model ANOVA with block, sculpin removal, trout removal and sculpin × trout interaction effects. We then directly analysed the overall composition of stickleback diets, using a multivariate analysis of variance (MANOVA) testing for block, sculpin, trout and sculpin × trout effects on arcsine square-root proportions of all prey categories. We analysed both raw frequencies of prey taxa and frequencies of functional groups: benthic cladocera, pelagic cladocera, benthic copepods, pelagic copepods, molluscs, benthic macroinvertebrates (insect larvae and Gammarus), pelagic macroinvertebrates, stickleback eggs and terrestrial/aerial insects (following Svanbäck & Bolnick 2007). We also evaluated prey-use differences between treatments using non-metric multidimensional scaling. All approaches yielded similar results, so we report the MANOVA results for functional groups.

Finally, we tested whether ecological release changed individual niche widths and the degree of individual specialization. We used mixed model ANOVAs to test for block, sculpin, trout and sculpin × trout effects on mean individual niche width (WIC), the among-individual variation (BIC) and two standardized measures of niche variation (WIC/TNW and *E*).

## 3. Results

### (a) Population-level effects of interspecific competition

Comparison of stickleback stomach contents (*n* = 825) with those of sculpin (*n* = 16) and trout (*n* = 20) confirms that there is dietary overlap among these species (electronic supplementary material). PS between stickleback and sculpin was 0.297, indicating that roughly a third of the prey consumed by stickleback are also prey for sculpin. This diet overlap appears low at first glance, but is only marginally less than the average diet overlap between individual sticklebacks in our study (PS = 0.382). We infer that interspecific competition between sculpin and stickleback is likely, but is weaker than intraspecific competition among stickleback. Stickleback also exhibited weak but non-zero diet overlap with trout (PS = 0.141).

Stickleback released from interspecific competitors had greater size-adjusted stomach content mass. Release from any single competitor had no significant effect on residual gut fullness (*p* = 0.819 and 0.829 for sculpin and trout, respectively; see the electronic supplementary material, table S1, for detailed statistical results). However, there was a significant sculpin × trout interaction (*p* = 0.004) owing to increase in gut fullness when both competitors were removed (electronic supplementary material, fig. S1). CI did not vary across treatments (all fixed effects *p* > 0.4). In comparison, Svanbäck & Bolnick (2007) found that intraspecific competition reduced stomach fullness, growth and condition, using a similar enclosure design and duration.

We observed no consistent change in sticklebacks’ overall diet distribution as a result of competitive release (MANOVA on arcsine-square-root-transformed diet proportions for each enclosure; block: *p* = 0.068; sculpin: *p* = 0.825; trout: *p* = 0.523; sculpin × trout interaction: *p* = 0.584). Note that this does not necessarily mean that, within a block, stickleback diets were similar across experimental treatments. Comparisons of particular pairs of enclosures (for instance, trout versus no trout within a block) generally do exhibit significant differences in prey composition (detailed results not shown). Rather, the non-significant MANOVA indicates that treatment-induced changes in prey use did not occur in a repeatable manner across blocks.

**Figure 2.**
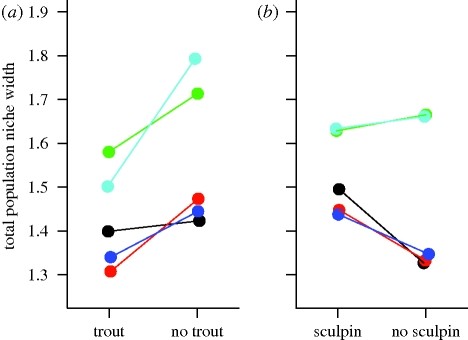
Effect of competitor removal on stickleback population total niche width (TNW). To visually represent the effect of release from trout, we calculated the average TNW with and without trout for a given block, averaging across sculpin treatments. Similarly, TNW with versus without sculpin are averaged across trout treatments within a block. For simplicity, we do not illustrate the non-significant sculpin × trout interaction. Lines connect competitor present versus absent results for a given block. Blocks are colour-coded to permit comparisons across (*a*) and (*b*), and with other figures.

We found a significant treatment effect on sticklebacks’ TNW (figure 2). Release from trout competition significantly increased stickleback TNW (*p* = 0.007), whereas sculpin and sculpin × trout effects were not significant (*p* = 0.286 and 0.147, respectively). To isolate the relative contributions of prey taxon richness versus evenness, we used multiple regression analysis to test whether TNW depends on richness (log of the number of prey taxa used in an enclosure) and/or evenness (TNW divided by richness). We found that variation in TNW arose from changes in evenness (*r*^2^ = 0.53; *p* = 0.0017) but not richness (*p* = 0.4985).

### (b) Individual-level effect of interspecific competition

All populations exhibited significant individual specialization (WIC/TNW averaged 0.518, *E* averaged 0.618; electronic supplementary material, table S2). Monte Carlo resampling confirmed that the observed diet variation among individuals was statistically significant (*p* < 0.001 in all samples for both indices) and therefore could not be explained by stochastic variation among individuals owing to limited numbers of prey per stomach (approx. 20 prey per fish on average).

**Figure 3.**
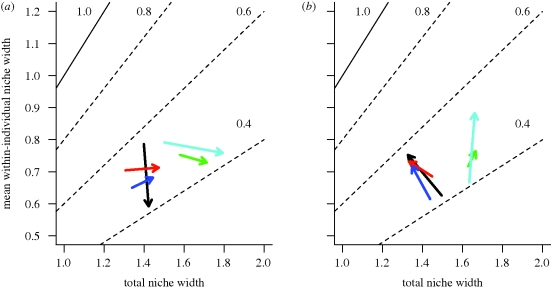
Effect of ecological release from (*a*) trout and (*b*) sculpin on stickleback population and individual niche widths. Release is plotted in a subset of the niche width space (explained in figure 1). Experimental effects are represented as vectors (one per experimental block). The vectors connect the mean (TNW, WIC) combination for competitor-present to competitor-absent treatments within a block, averaging across the other competitor treatments. As in figure 1, dotted lines represent isoclines of WIC/TNW, with individual specialization increasing as WIC/TNW declines from 1.0 towards zero. Blocks are colour-coded to correspond with other figures.

**Figure 4.**
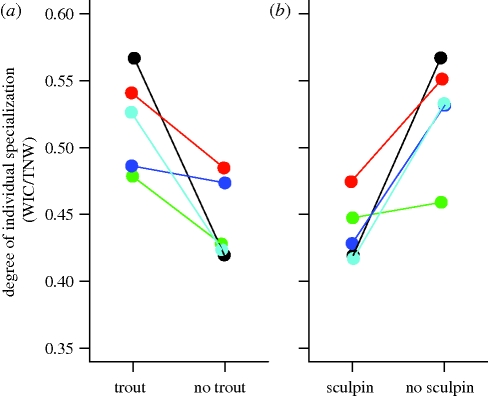
Effect of competitor removal on the degree of individual specialization in stickleback (WIC/TNW). When WIC/TNW = 1, individuals have the same niche breadth as the population as a whole. As WIC/TNW gets smaller, individuals are increasingly specialized relative to their population and between-individual variation is proportionally larger. Each point is the average value for a given block of enclosures, averaging across the other competitor treatments. Lines connect competitor-present versus competitor-absent results for a given block. Blocks are also colour-coded to permit comparisons across (*a*) and (*b*) and with other figures. We do not illustrate the non-significant interaction between trout and sculpin removal.

Release from interspecific competitors altered how TNW was partitioned into within- versus between-individual diversity (figure 3). Although sculpin release had no effect on TNW, it did increase individual niche breadth (WIC; *p* = 0.003), and decreased between-individual variation (BIC; *p* = 0.022). The opposing changes in WIC and BIC cancelled each other out, explaining the lack of a sculpin effect on TNW. As a result, release from sculpin competition led to reduced individual specialization in stickleback (increased WIC/TNW, *p* = 0.0019; figure 4). These results closely match our ‘individual release’ scenario (figure 1*c*). The increase in WIC/TNW is corroborated by decreased pairwise diet dissimilarity among individuals (*E*; sculpin effect, *p* = 0.0279). We found no significant sculpin × trout interactions (WIC: *p* = 0.147; BIC: *p* = 0.077; WIC/TNW: *p* = 0.287). There was a weak tendency towards an interaction effect for BIC: sculpin release had no significant effect on BIC when trout were present, but a strong negative effect on BIC when trout were absent.

Stickleback niche expansion during trout release was predominantly a result of increased between-individual variation (BIC, *p* = 0.01), consistent with the NVH. Trout had no consistent effect on WIC (*p* = 0.293; figure 3). Because TNW = WIC + BIC, static WIC and increased BIC yielded higher TNW. In stark contrast to sculpin effects, trout release thus caused increased individual specialization, measured either by decreased WIC/TNW (*p* = 0.016; figure 4) or increased *E* (*p* = 0.0378).

In principle, changes in individual niche width during competitive release might simply be an artefact of using cross-sectional stomach content analysis. If individuals consume more prey items following ecological release, then stomach contents are likely to contain a higher prey diversity, leading to apparent (but not biologically relevant) increases in individual niche width. We reject this artefact because we observed no significant trout or sculpin effect on the mean *per capita* number of prey consumed (*p* > 0.4 in both cases), so treatment effects on diet cannot reflect prey count differences.

## 4. Discussion

Ecological release from interspecific competition has long been thought to allow population niche expansion (Van Valen 1965; Roughgarden 1972; Grant & Price 1981; Feinsinger & Swarm 1982; Taper & Case 1985; Robinson & Wilson 1994; Losos & de Queiroz 1997; Robinson *et al*. 2000; Svanbäck *et al*. 2008). By experimentally manipulating interspecific competition, we found mixed support for competitive release of population niche width. Release from trout competition induced a statistically significant 10 per cent increase in stickleback TNW, owing to increased evenness of prey use rather than the addition of novel prey. In contrast, sculpin had no significant effect on stickleback TNW; if anything the trend was towards decreased TNW. Thus, we found support for the idea of ecological release from one of the two competitor species (despite their similar effects on stickleback foraging success and CI). However, focusing on populations’ TNW masks some additional responses to competitive release.

### (a) Ecological release for individuals

As in previous studies of stickleback diets, we found substantial individual specialization in three-spine stickleback. Average individual niche widths (WIC) within enclosures ranged from 35 to 65 per cent of the population's TNW (electronic supplementary material, table S2). Similarly, mean pairwise diet dissimilarity between individuals ranged from *E* = 0.39 to 0.71. This degree of individual specialization was significantly greater than expected under the null hypothesis of a single shared prey distribution, and was comparable with results of other studies of stickleback (Bolnick 2004; Svanbäck & Bolnick 2007; Bolnick *et al*. 2008; Snowberg & Bolnick 2008; Bolnick & Paull 2009).

Notably, strong diet variation was maintained even in constrained enclosures (10 m^2^), where all fish can readily swim between all possible foraging sites in a matter of seconds. Indeed, individual specialization was actually stronger in enclosures than in neighbouring wild-caught fish (electronic supplementary material, fig. S2), perhaps owing to slightly elevated intraspecific competition within the enclosures (Svanbäck & Bolnick 2007). Thus, spatial segregation of prey (at a scale greater than an individual fish's daily cruising range) is not the primary force driving niche variation among individual sticklebacks. Rather, the diet variation appears to be a consequence of individuals’ persistent prey preferences, at least partly owing to specialization on fine-scale microhabitats (Bolnick *et al*. in review) and morphological variation among individuals (Araújo *et al*. 2008).

Among-individual niche variation means that individuals’ responses to ecological release may not match patterns of whole-population response to release (figure 1). Whereas whole-population competitive release was seen for trout but not sculpin, at the individual level the opposite was true (release was seen for sculpin but not trout). In trout, increased population niche width arose via increased between-individual variation but constant individual niche width, consistent with the NVH (Van Valen 1965). We draw two major conclusions from these results. First, trout competition opposes the diversifying effect of intraspecific competition (Svanbäck & Persson 2004; Svanbäck & Bolnick 2007), supporting the classical model of a tension between inter- and intraspecific competition. Second, our result highlights the discrepancy between individual- and population-level responses to ecological interactions.

Sculpin had a very different effect on stickleback diets than trout did. Although there was no support for whole-population ecological release from sculpin, sculpin did modify how individual sticklebacks partitioned the available resources. Release from sculpin competition increased individual niche widths by an average of 20 per cent, but decreased between-individual variation by an equivalent amount. Thus, population niche width (TNW = WIC + BIC) did not change, because individual niche expansion does not involve adoption of novel prey at the population level (e.g. Svanbäck & Bolnick 2005). These results match the ‘individual release hypothesis’ (figure 1*b*), in which changes in prey availability cause individuals to increase their reliance on prey that they previously ignored, but which were used by other conspecifics.

### (b) Ecological release on behavioural time scales

Ecological release has generally been viewed as an evolutionary process. Species released from competition are subject to directional or disruptive natural selection on trophically relevant phenotypes, driving increased niche width and/or phenotypic variance over the course of multiple generations (Lister 1976; Robinson & Wilson 1994; Losos & de Queiroz 1997; Robinson *et al*. 2000). However, our experiment shows that ecological release and among-individual diversification can also occur rapidly within a generation. One possible explanation for this rapid change could be that our experimental treatment induced very strong viability selection that induced rapid evolutionary changes in resource use. We reject this hypothesis, because we observed no systematic between-treatment difference in survival (average of 91% recapture; *p* = 0.84, 0.60, 0.49 for effects of trout, sculpin or their interaction, respectively). We also observed no significant difference in phenotypic means or variances among treatments (*p* > 0.1 for all treatment and block effects on mean or variance in fish size, gape or gill raker traits).

The more probable explanation for rapid niche changes is that individuals’ foraging behaviour changed in response to short-term changes in prey availability. Similar rapid behavioural shifts were also observed in stickleback responding to intraspecific competition (Svanbäck & Bolnick 2007). Optimal foraging theory predicts that individuals modify their behaviour to maximize their expected fitness as prey availability changes (Stephens & Krebs 1986; Sih & Christensen 2001). Adaptive foragers are thus expected to exhibit rapid behavioural diet shifts that should mirror long-term evolutionary expectations for phenotypic selection. Such prey-switching behaviours create temporal shifts in resource use that can rapidly restructure food webs, and thereby alter the dynamics of entire ecological communities (Kondoh 2003). However, our results also emphasize that foraging behaviour is only flexible within limits, as individual niche widths were constrained (during trout release) and were consistently narrower than population niche widths. Models of flexible foraging in food webs need to begin to incorporate such constraints.

### (c) Contrasting effects of two competitors

Trout and sculpin removal induced diametrically opposite forms of ecological release in stickleback. Trout release caused population (but not individual) niche expansion, while sculpin release caused individual (but not population) niche expansion. At present, we are unable to determine the mechanistic reasons for this disparity. A mechanistic analysis would have to evaluate the effects of each competitor on (i) the availability of stickleback prey and (ii) patterns of prey selectivity by the stickleback. The former data would have required intensive sampling of benthic and pelagic prey throughout the experiment to determine specific changes in prey availability. We chose not to collect these data because repeated prey sampling would have been excessively disruptive to the main experiment. The latter would require direct observations of individuals’ foraging preferences in the field enclosures, as a function of their phenotypes and the changing availability of prey.

While a mechanistic analysis is far beyond the scope of the present study, we can offer several insights into differences between trout and sculpin as competitors with stickleback. First, although both species exhibited dietary overlap with stickleback, this overlap differed in magnitude. Stickleback/sculpin diet overlap was 78 per cent as large as intraspecific diet overlap among stickleback individuals, compared with 37 per cent for stickleback/trout overlap. These different capacities for interspecific competition might be exacerbated by differences in metabolic rates, interference interactions, etc. Despite these probable differences, we found no consistent between-treatment differences in the taxonomic or functional composition of stickleback stomach contents at the end of the experiment. However, for a MANOVA to detect a competitor effect on stickleback diet composition, the competitor would have had to induce the same type of diet shift in each block of enclosures. Although stickleback consistently increased evenness of prey use in the absence of trout, replicates differed as to which particular prey taxa were responsible for the increased evenness, perhaps owing to spatial heterogeneity among blocks. This heterogeneity could also occur if there is individual specialization within the competitor species, and such trout placed in enclosure A ate different prey than trout in enclosure B. Unfortunately, we had too few competitor recaptures to evaluate this effect.

### (d) Conclusions

This study presents one of the few experimental tests of whether ecological release from competitors can lead to niche expansion (Holmes 1973; Colwell & Fuentes 1975; Pacala & Roughgarden 1985; Persson & Hansson 1999). Such release has long been accepted by ecologists on the basis of comparisons of populations in different competitive environments (Van Valen 1965; Grant 1966; Diamond 1971; Terborgh & Faaborg 1973; Kohn 1978; Feinsinger & Swarm 1982; Dayan & Simberloff 1994; Robinson & Wilson 1994; Holbrook & Schmitt 1995; Trewby *et al*. 2007), and is presumed to play a central role in adaptive radiations (Losos & de Queiroz 1997). Our experimental results support the conclusions of these comparative studies, with an unsurprising caveat that not all competitor species will have the same effects. More importantly, we demonstrate that there are multiple ways in which ecological release from competition can alter a population's resource use. Depending on the competitor examined here, ecological release either involved population niche expansion according to the NVH, or the individual release hypothesis. These contrasting responses to ecological release are made possible by the fact that among-individual variation decouples individual and population niche widths. We do not yet fully understand why individual and population niche widths are decoupled, nor do we understand the mechanistic reasons why different competitors drove such distinct forms of ecological release. Nevertheless, our results do make it clear that to understand the ecological and evolutionary consequences of changes to a species's niche, it will be necessary for future ecological models to more carefully distinguish between the behaviour of individuals and their population as a whole.
